# Generation of Ammonia in a Pulsed Hollow Cathode Discharge Operated in an Ar/H_2_/N_2_ Gas Mixture Detected by Fourier Transform Infrared

**DOI:** 10.1021/acssuschemeng.4c08054

**Published:** 2024-11-15

**Authors:** Rainer Hippler, Martin Cada, Antonin Knizek, Martin Ferus, Zdenek Hubicka

**Affiliations:** † Institute of Physics, Czech Academy of Sciences, Na Slovance 2, 18200 Prague, Czech Republic; ‡ Institut für Physik, Universität Greifswald, Felix-Hausdorff-Strasse 6, 17489 Greifswald, Germany; § J. Heyrovsky Institute of Physical Chemistry, Czech Academy of Sciences, Dolejskova 2155/3, 18223 Prague, Czech Republic

**Keywords:** ammonia synthesis, hollow cathode discharge, ion mass spectrometry

## Abstract

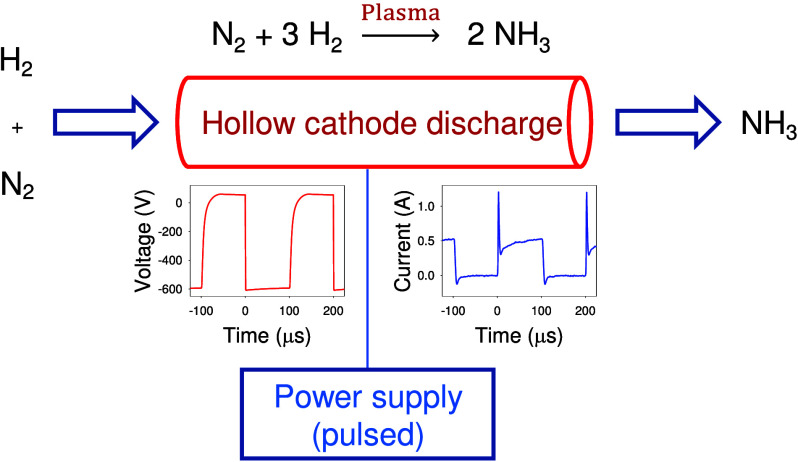

A hollow cathode discharge with a copper nickel cathode (Cu50Ni50) was operated in an Ar/H_2_/N_2_ gas mixture. Optical emission spectroscopy revealed the formation of NH radicals, which serve as precursors for NH_3_ formation. Ion mass spectrometry showed the formation of NH_3_^+^ and NH_4_^+^ ions indicating NH_3_ formation. Gas samples taken at the exhaust of the vacuum system were analyzed by Fourier transform infrared spectroscopy. Clear evidence for NH_3_ formation was obtained from these measurements.

## Abstract 2

A pulsed hollow cathode discharge was employed for the plasma-assisted generation of green ammonia.

## I Introduction

Ammonia (NH_3_) is an important chemical that can be used for fertilizer production, for energy storage, as an alternative fuel in transportation, and in a future hydrogen-based economy.^1−11^ Much of the required NH_3_ is currently produced by making use of the Haber–Bosch process.^12,13^ The Haber–Bosch process converts atmospheric nitrogen (N_2_) and molecular hydrogen (H_2_) to NH_3_ by a reaction which requires a catalyst, high temperatures of 650–750 K, and high pressures of 15–30 MPa.^14−18^ The process was invented by Fritz Haber in 1905 and further developed by Carl Bosch to an industrial scale.^12,13^ The Nobel prize in chemistry was awarded to Fritz Haber in 1918 “for the synthesis of ammonia from its elements” and to Carl Bosch (together with Friedrich Bergius) “in recognition of their contributions to the invention and development of chemical high pressure methods”.^19^

At standard conditions, NH_3_ has a melting point of 195.4 K (−77.7 °C), a boiling point of 239.8 K (−33.35 °C), and a gas density of 0.77 kg/m^3^.^20^ Compared to H_2_ with a boiling point of 20.27 K (−252.8 °C), NH_3_ is easily liquefied and stored. Ammonia has a large energy density of 22.5 kJ/g and a large hydrogen content of 75 atom %.^21^ Global industrial ammonia production is currently approaching 200 Mt/year. NH_3_ production presently contributes about 2% (8.6 EJ) to the global energy consumption^22^ and about 1.3% (450 Mt) to the global CO_2_ emission. As such, there is some need for production processes that consume less energy and cause fewer emissions.

Plasma-assisted NH_3_ synthesis is receiving renewed interest as an alternative for the Haber–Bosch process.^10,16,23−30^ Nonthermal low-temperature plasmas are characterized by *hot* electrons and comparatively *cold* atoms, molecules, and ions.^31,32^ Plasmas are readily operated with renewable energy^33−35^ from, e.g., solar cells and/or windmills, and provide better energy efficiency, lower capital cost, and, in combination with a catalyst, a larger catalyst operation time.^24^

In the present communication, we employ a pulsed hollow cathode (PHC) discharge to confine the plasma. The PHC discharge is operated in pulsed mode with a pulse length of 100 μs and a repetition frequency of 5 kHz (duty cycle 50%) in an Ar + N_2_ + H_2_ gas environment with the gas flow directed through the hollow cathode (HC). Ar gas was added to stabilize the discharge. The plasma is sustained by electrons that undergo a pendulum motion inside the cathode.^36−42^ As a novel aspect, we mention that the cathode can be made from metals with suitable catalytic properties to enhance the reaction rates.^24,43−45^ In this pilot study, we utilize a cathode made from Cu50Ni50 alloy, which is known to have catalytic and photocatalytic properties.^46^ Other cathode materials will be investigated in future studies. Generation of ammonia is detected with the help of Fourier transform infrared (FTIR) spectroscopy.

## II Experiment

**Figure 1:**
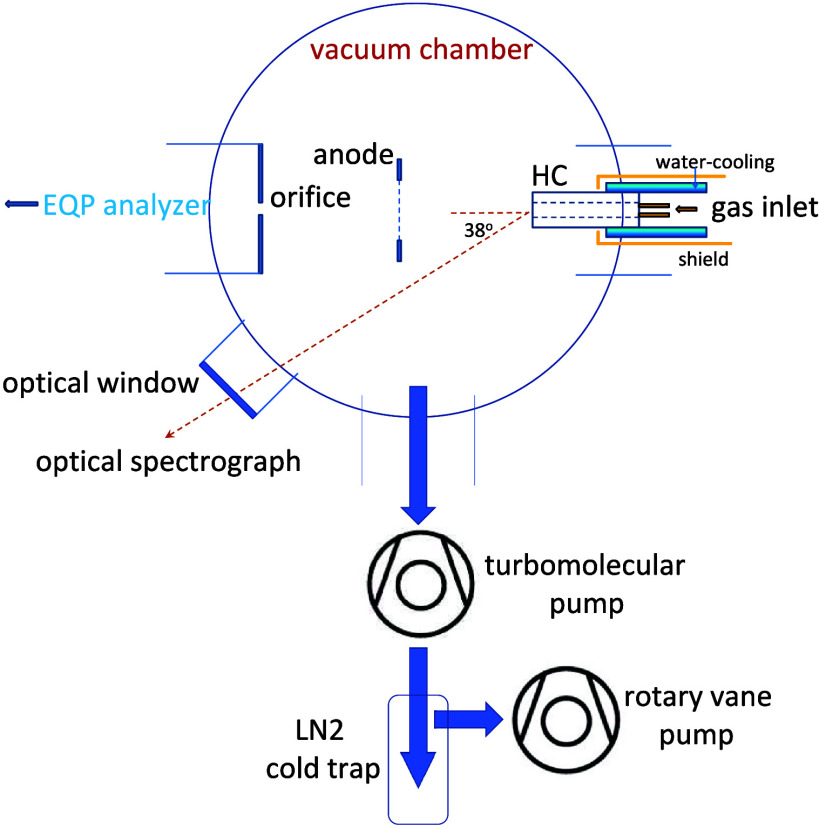
Experimental setup (schematic).

The experiment, as shown in Figure 1, employs a cylindrical HC inside a vacuum chamber.^46,47^ The chamber is evacuated by a combination of a rotary and a turbomolecular pump (base pressure of 1 × 10^–4^ Pa). The HC is manufactured from a copper nickel (Cu50Ni50) alloy with 99.95% purity; it has a length of 40 mm, an outer diameter of 12 mm, and an inner diameter of 5 mm. The HC is water-cooled; however, it protrudes out of the cooling holder, and its open end heats up to high temperatures.^48,49^ In order to stabilize the discharge, an external ring anode in a distance of 100 mm from the HC is employed.^50^ Argon (purity 99.999%), hydrogen (purity 99.995%), and nitrogen (purity 99.999%) gases are introduced to the vacuum chamber through the HC. Three mass flow controllers are employed. The Ar gas flow rate is set to 200 sccm resulting in a chamber pressure of 2.1 Pa. Typical hydrogen and nitrogen gas flow rates are in the range 10–50 sccm.

The HC is powered by a direct-current power supply (Advanced Energy MDX-500) in combination with a home-built power switch enabled during pulsed operation. A commercial arbitrary waveform generator (OWON AG 1022) is employed to set the repetition frequency *f* = 5 kHz and the pulse length *T*_on_ = 100 μs. The PHC discharge is operated in a current control mode with a mean discharge current of 0.25 A and a typical discharge power of about 80 W.

**Figure 2:**
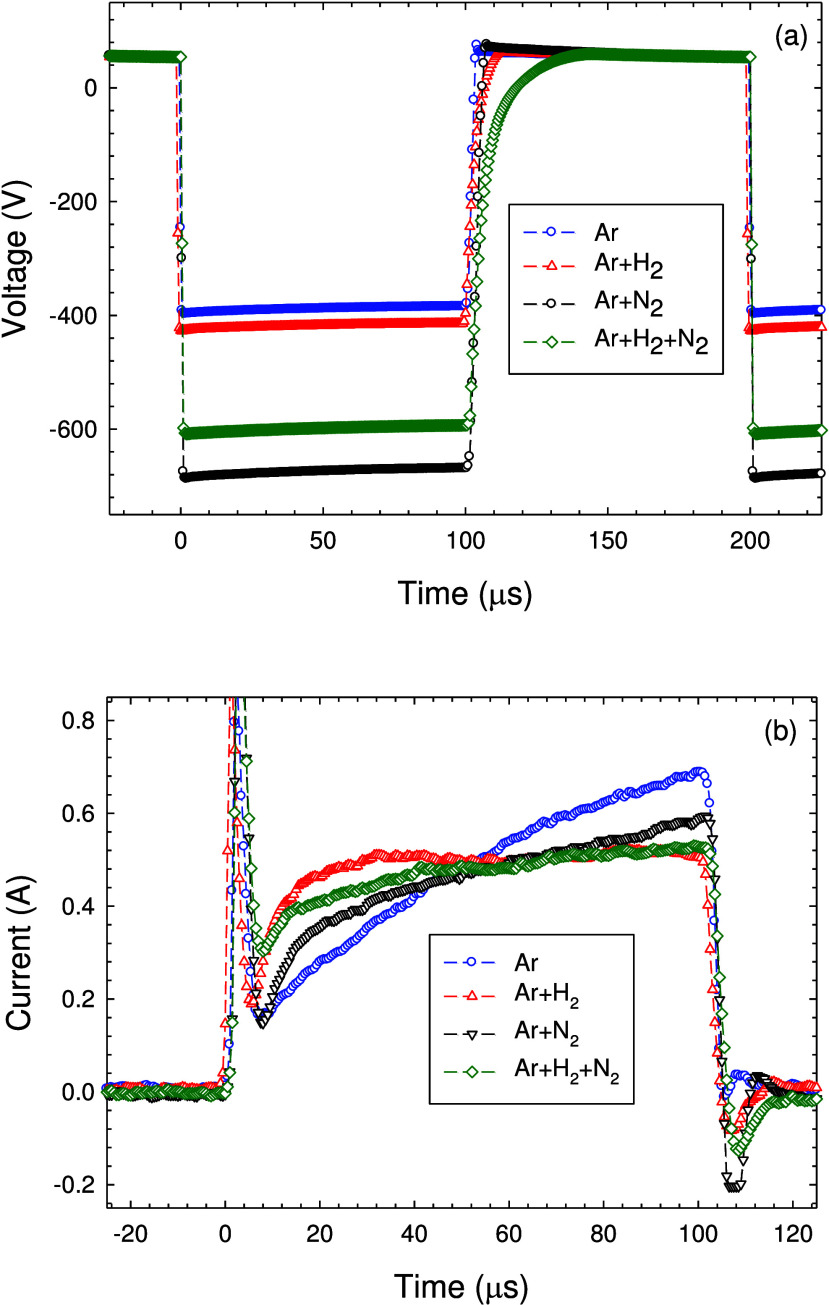
(a) Discharge voltage and (b) discharge current versus time. Ar gas flow rate = 200 sccm, H_2_ gas flow rate = 100 sccm, and N_2_ gas flow rate = 100 sccm. Mean discharge current = 0.25 A.

The time evolution of discharge voltage and discharge current is shown in Figure 2 for a PHC discharge operated in pure Ar and in Ar + H_2_, Ar + N_2_, and Ar + H_2_ + N_2_ gas mixtures. The same mean discharge of 0.25 A is used. The (negative) discharge voltage shows a rapid decrease, which is controlled by the mean discharge current and depends on the employed gas mixture. The smallest initial discharge voltages of −390 and −420 V are required for Ar and Ar + H_2_, respectively, whereas much larger voltages of −605 and −685 V are required to ignite the Ar + H_2_ + N_2_ and Ar + N_2_ gas mixtures, respectively. The temporal evolution of the discharge current for the different gas mixtures also shows significant differences. For example, after an initial overshooting right at the beginning of the pulse, the discharge monotonously increases during the pure Ar case but quickly saturates for the Ar + H_2_ case.

Energy-resolved mass spectrometry is performed with a commercial Hiden EQP 1000 mass/energy analyzer (Hiden Analytical Ltd., U.K.).^51,52^ The instrument is mounted opposite to the HC and anode at a distance of 120 mm from the anode.^50,53,54^ Optical emission spectroscopy (OES) is carried out with a Shamrock SR500D spectrometer (focal length = 500 mm) equipped with an iCCD detector (iStar DH334T18UE3, Andor Technology, Belfast, Northern Ireland). The spectrometer is equipped with three gratings and with a filter wheel in front of the entrance slit to suppress second-order lines. An optical fiber connected to the entrance slit (slit width = 20 μm) of the spectrometer is installed outside the vacuum chamber at an angle of 38° with respect to the cathode axis.

Ex situ FTIR spectoscopy is employed to analyze the exhaust gas from the plasma chamber. Exhaust gas constituents are trapped in a liquid-nitrogen-cooled glass container that is inserted into the exhaust line between the turbomolecular pump and rotary pump. After removal from the exhaust line, the cold gas container is connected to a glass cell with ZnSe windows and an optical path of 28 cm. The cell is inserted into a high-resolution FTIR spectrometer (Bruker IFS 125HR, Bruker GmBH, Karlsruhe, Germany), which is operated in the mid-infrared region between 600 and 6000 cm^–1^. The infrared spectra are used to identify the molecular composition of the sample and to obtain the partial pressures for quantification. Details of the experiment are described elsewhere.^47^

## III Results and Discussion

### A OES

**Figure 3:**
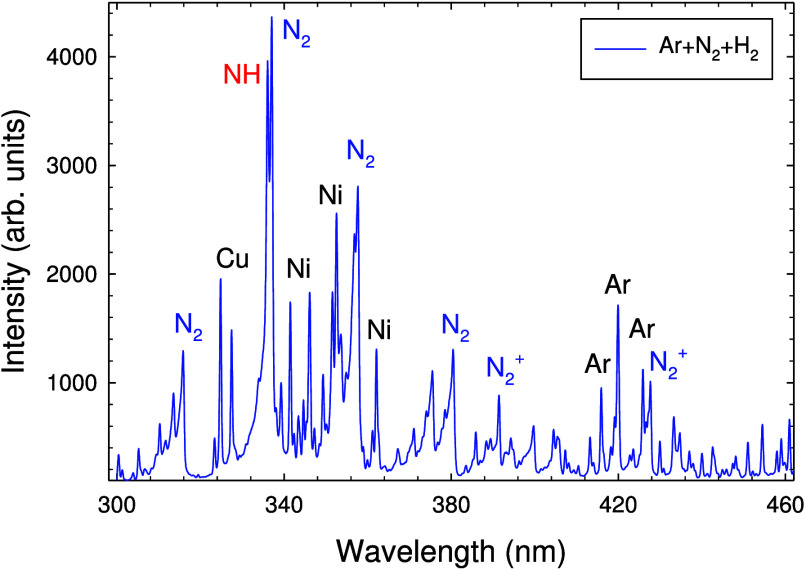
OES spectrum in the wavelength range of 300–480 nm obtained with the Ar + H_2_ + N_2_ gas mixture showing Ar I, Cu I, and Ni I lines and molecular NH, N_2_, and N_2_^+^ emission bands. Optical grating = 600 lines/mm. Ar gas flow rate = 200 sccm, H_2_ gas flow rate = 20 sccm, and N_2_ gas flow rate = 20 sccm. Discharge current = 0.25 A.

OES was carried out in the spectral region from 200 to 1000 nm. No NH_3_ emission bands exist in this wavelength region.^55^Figure 3 shows the spectral region from 300 to 460 nm. In this range, several molecular N_2_, N_2_^+^, and NH emission bands as well as atomic (Cu I, Ni I, and Ar I) and ionic (Ar II) emission bands are observed. Most notable are the Cu I resonance lines at 327.8 and 327.4 nm,^56^ the Ni I lines at 339.3, 341.5, 345.8, 345.8/346.2, 349.3, 351.5, 352.5, 356.6, and 361.9 nm, and the Ar I lines at 415.9, 419.8/420.1, and 426.0 nm.^56^ Several Ar II (Ar^+^) lines are detected in the range from 427.5 to 480 nm. Several molecular bands appear in the spectrum, most notably from the N_2_(C ^3^Π_u_–B^3^Π_g_) second positive system at 315.9, 337.1, 375.5, and 380.5 nm^57−60^ and the N_2_^+^(B^2^Σ_u_^+^–X ^2^Σ_g_^+^) bands at 391.4 and 427.8 nm.^60^

**Figure 4:**
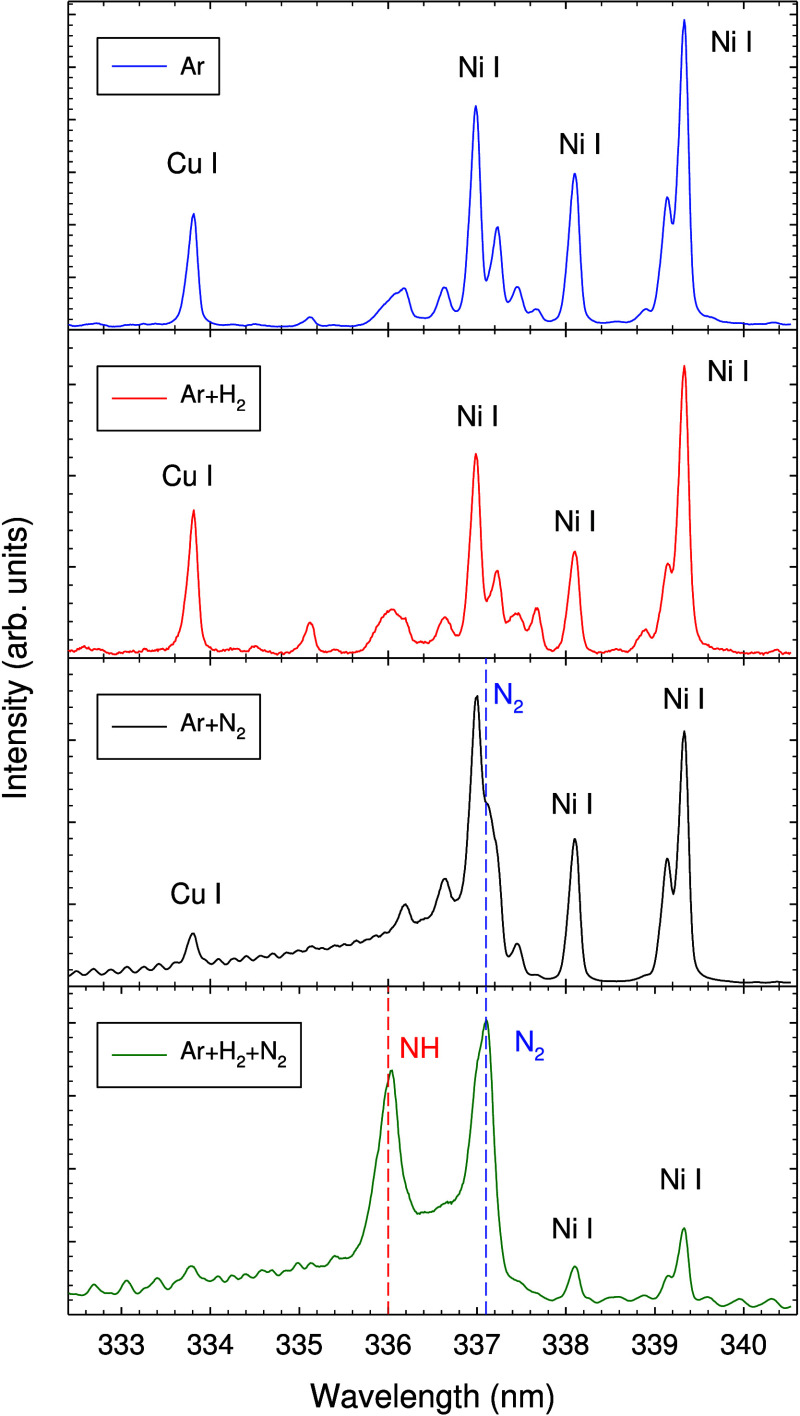
OES spectrum in the wavelength range of 333–340 nm with different gas mixtures (Ar, Ar + H_2_, Ar + N_2_, Ar + H_2_ + N_2_) showing NH (336.0 nm) and N_2_ (337.1 nm) emission bands. Optical grating = 2400 lines/mm. Ar gas flow rate = 200 sccm, H_2_ gas flow rate = 20 sccm, and N_2_ gas flow rate = 20 sccm. Mean discharge current = 0.25 A.

High-resolution OES spectra of the wavelength range 333–340 nm are displayed in Figure 4 for different gas mixtures. The OES spectrum of pure Ar shows a Cu I line at 333.8 nm and several Ni I lines at 337.0, 338.1, 339.1, and 339.3 nm.^56^ A similar spectrum is obtained for the Ar + H_2_ gas mixture. The same metallic (Cu I and Ni I) lines also appear in the spectrum for the Ar + N_2_ gas mixture. In addition, a N_2_ band at 337.1 nm appears and partly overlaps with the Ni I line at 337.0 nm. The OES spectrum obtained with the Ar + H_2_ + N_2_ gas mixture shows the appearance of a new peak at 336.0 nm, which is attributed to the NH(A^3^Π–X^3^Σ^–^) band.^61−63^ It indicates the formation of NH radicals, which are intermediates for NH_3_ formation.

Without catalyst, the main plasma-assisted reactions include dissociation, excitation, and ionization of molecules by sufficiently energetic electrons. In a plasma environment containing H_2_ or N_2_ molecules, dissociation of molecules by electron impact gives rise to the formation of H or N radicals. Reactions of metastable Ar* atoms may as well contribute to the dissociation of N_2_ and H_2_.^64,65^ Subsequent reactions of H or N with N_2_ or H_2_ molecules lead to the formation of NH radicals via, e.g.,^10,25,66,67^
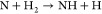
1or
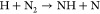
2Of these, reaction 1 dominates, while reaction 2, although highly endothermic, has a rather small rate coefficient.^68^

### B Mass Spectrometry

**Figure 5:**
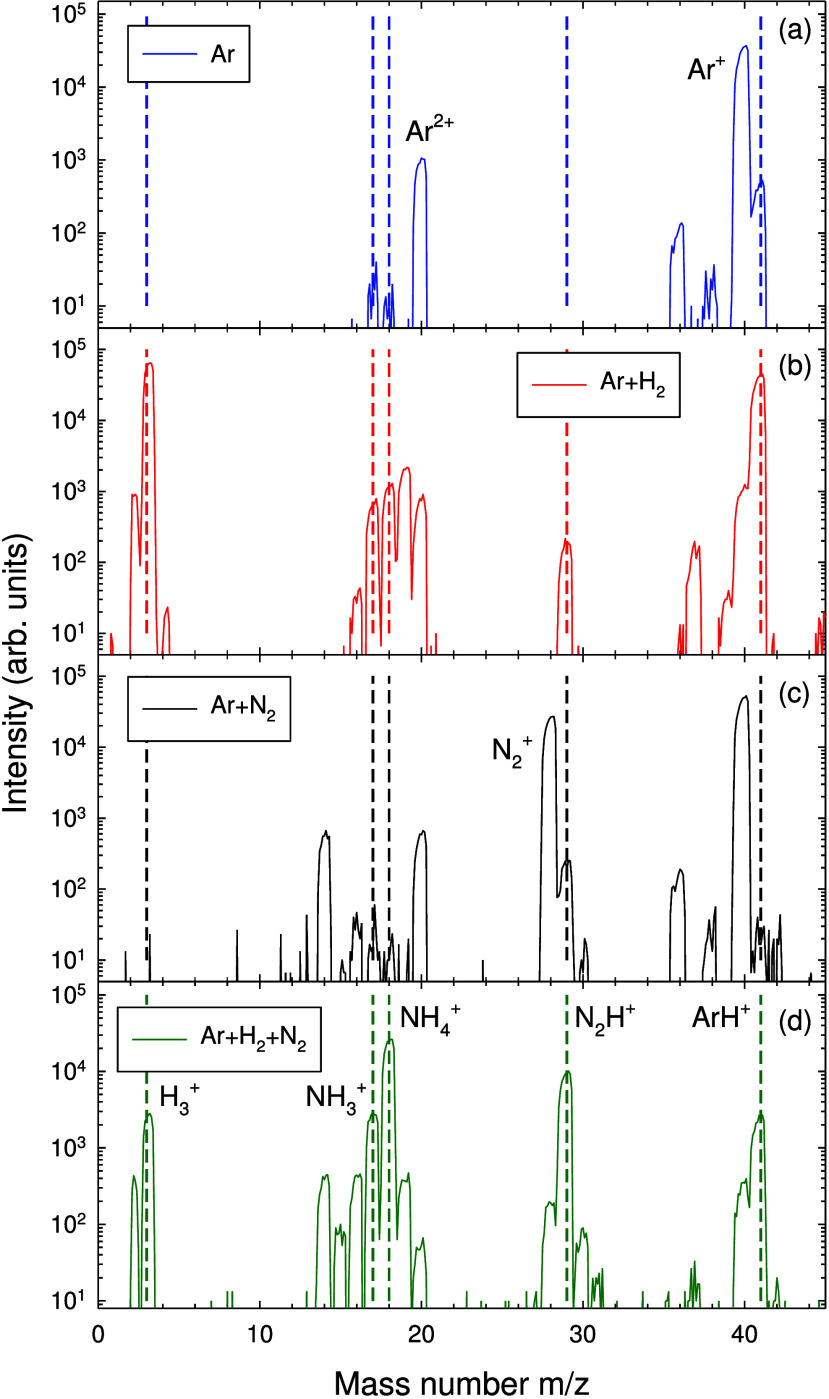
Ion mass spectra of a PHC discharge with a Cu50Ni50 nozzle operated in (a) Ar, (b) Ar + H_2_, (c) Ar + N_2_, and (d) Ar + H_2_ + N_2_ gas mixtures. Pulse length = 100 μs, repetition frequency = 5 kHz, anode voltage = +60 V, and mean discharge current = 0.25 A. Argon gas flow rate = 200 sccm, hydrogen gas flow rate = 100 sccm, and oxygen gas flow rate = 100 sccm, Ar gas pressure *p* = 2.1 Pa. Detected ion energy = 40 eV. Vertical dashed lines indicate the mass positions of H_3_^+^ (*m*/*z* 3), NH_3_^+^ (*m*/*z* 17), NH_4_^+^ (*m*/*z* 18), N_2_H^+^ (*m*/*z* 29), and ArH^+^ (*m*/*z* 41).

Typical ion mass spectra for a PHC discharge operated in different gas mixtures are displayed in Figure 5. The mass spectrum from pure Ar is dominated by Ar^+^ (*m*/*z* 40) and Ar^2+^ (*m*/*z* 20) ions, where *m* and *z* are ion mass and charge numbers, respectively. The mass spectrum from the Ar + H_2_ gas mixture additionally displays a strong H_2_^+^ (*m*/*z* 2) signal and several mass peaks related to impurity ions that are associated with water, e.g., OH^+^ (*m*/*z* = 17), H_2_^+^O (*m*/*z* 18), and H_3_^+^O (*m*/*z* 19);^69^ these ions presumably form via the interaction of H radicals with oxidized parts of the chamber’s walls. The Ar^+^ mass peak is significantly reduced, and a new peak originating from ArH^+^ ions (*m*/*z* 41) appears. The mass spectrum from the Ar + N_2_ gas mixture compared to pure Ar gas shows several additional mass peaks that are related to nitrogen, e.g., from N^+^ (*m*/*z* 14) and N_2_^+^ (*m*/*z* 28). Last, the ion mass spectrum from the Ar + H_2_ + N_2_ gas mixture is dominated by two ion mass peaks at *m*/*z* 17 and 18, which are attributed to the formation of ammonia (NH_3_^+^) and ammonium (NH_4_^+^) ions.^69,70^ Collisions of plasma ions, e.g., H_2_^+^, N_2_^+^, and N_2_H^+^ with NH_3_ molecules efficiently contribute to NH_3_^+^ and NH_3_^+^ formation.^71,72^ Ion mass distributions are to a large extent determined by plasma chemical ion–molecule reactions and, in particular, the dominance of NH_4_^+^ ions indicates that neutral NH_3_ molecules are produced in appreciable concentrations.^71^ We briefly mention the strong interaction of N_2_ molecules or of argon atoms with, e.g., H_2_^+^ ions which is responsible for the formation of N_2_H^+^ (*m*/*z* 29) or ArH^+^ (*m*/*z* 41) ions, respectively.^70^

### C Infrared (FTIR) Spectroscopy

**Figure 6:**
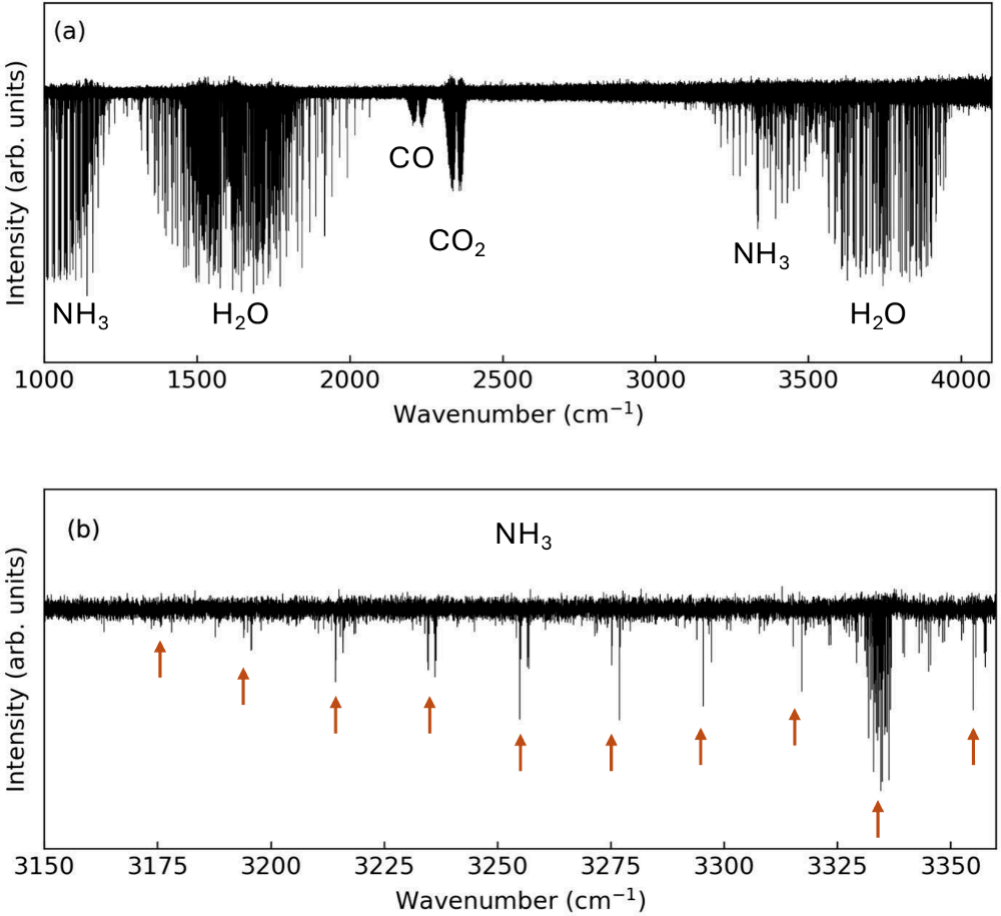
FTIR of a PHC discharge with a Cu50Ni50 nozzle. Panel a shows the measured FTIR spectrum in the range of 1000–4100 cm^–1^. The spectrum shows absorption bands of ammonia, water (H_2_O), carbon monoxide (CO), and carbon dioxide (CO_2_). The presence of H_2_O, CO, and CO_2_ is attributed to the experimental background. Panel b shows the details of the ammonia ν_1_ symmetric stretch absorption band at 3337 cm^–1^. The main NH_3_ absorption lines are indicated by red arrows. Sampling time = 75 min. Pulse length = 100 μs, repetition frequency = 5 kHz, and mean discharge current = 0.15 A. Argon gas flow rate = 200 sccm, nitrogen gas flow rate = 10 sccm, hydrogen gas flow rate = 30 sccm, and gas pressure *p* = 2.3 Pa.

The formation of NH_3_ molecules is further confirmed by FTIR spectroscopy using gas samples which are taken from the exhaust line of the vacuum system. Figure 6 displays FTIR spectra obtained with an argon gas flow rate of 200 sccm, nitrogen gas flow rate of 10 sccm, and a hydrogen gas flow rate of 30 sccm. Ammonia has a melting point of 195.4 K and, hence, is, readily captured by the liquid-nitrogen-cooled surface.^20^Figure 6a presents the overall spectrum with shows clear absorption bands of ammonia as well as background gases, which are H_2_O, CO_2_, and CO. Figure 6b shows a zoom-in of the ammonia symmetric stretch absorption band centered at 3337 cm^–1^. The total volume of the sampled gas after 75 min of discharge operation is about 8.4 cm^3^ (at standard conditions). FTIR analysis shows that about 59% of the collected gas is NH_3_ and that the remaining 41% are mainly water (H_2_O). It appears likely that water is produced via the interaction of hydrogen species with oxygen-covered or oxygen-containing surfaces. The exact origin of the background gases is not known, however.

Among the possible plasma chemical reactions, collisions of NH radicals with H_2_ molecules, e.g.,
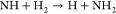
3followed by
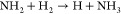
4eventually lead to the formation of NH_3_ molecules via plasma chemical reactions in the gas phase.^10,66,73^ In addition to the processes taking place in the plasma bulk, surface-based reactions of H, NH, NH_2_, and other radicals with H_2_ or N_2_ molecules adsorbed on the cathode surface may contribute to NH_3_ formation.^74,75^ If so, the material of the surface and its catalytic properties should have a significant influence on NH_3_ formation.^23,24,43,76^ An order of magnitude improvement through the use of a suitable catalyst appears feasible.^77−79^ The use of different cathode materials is planned in future investigations.

## IV Conclusions

Plasma chemical reactions in a PHC discharge with an H_2_ + N_2_ + Ar gas mixture are investigated. The formation of NH radicals as an intermediate step of NH_3_ formation is demonstrated with the help of optical spectroscopy. Formation of NH_3_^+^ and NH_4_^+^ ions indicating NH_3_ generation is observed using ion mass spectrometry. The generation of ammonia is confirmed by FTIR spectroscopy. The role played by the employed CuNi nozzle is not investigated yet and, with respect to the eventual catalytic properties of the cathode, remains a future issue.

## Data Availability

Data will be made available upon reasonable request and via https://doi.org/10.5281/zenodo.14165349.
